# Advancing Nanoscale Copper Deposition Through Ultrafast-Laser-Activated Surface Chemistry

**DOI:** 10.3390/nano15110830

**Published:** 2025-05-30

**Authors:** Modestas Sadauskas, Romualdas Trusovas, Evaldas Kvietkauskas, Viktorija Vrubliauskaitė, Ina Stankevičienė, Aldona Jagminienė, Tomas Murauskas, Dainius Balkauskas, Alexandr Belosludtsev, Karolis Ratautas

**Affiliations:** Center for Physical Sciences and Technology, Savanoriu Ave. 231, LT-02300 Vilnius, Lithuania; modestas.sadauskas@ftmc.lt (M.S.); romualdas.trusovas@ftmc.lt (R.T.); viktorija.vrubliauskaite@ftmc.lt (V.V.); ina.stankeviciene@ftmc.lt (I.S.); aldona.jagminiene@ftmc.lt (A.J.); tomas.murauskas@ftmc.lt (T.M.); alexandr.belosludtsev@ftmc.lt (A.B.)

**Keywords:** direct laser writing, submicron copper circuits, Bessel beam, glass metallization, ultrashort-pulse laser, electroless copper plating, silver ion activation, transparent electronics, high-density interconnects, lithography-free microfabrication

## Abstract

Direct-writing submicron copper circuits on glass with laser precision—without lithography, vacuum deposition, or etching—represents a transformative step in next-generation microfabrication. We present a high-resolution, maskless method for metallizing glass using ultrashort pulse Bessel beam laser processing, followed by silver ion activation and electroless copper plating. The laser-modified glass surface hosts nanoscale chemical defects that promote the in situ reduction of Ag^+^ to metallic Ag^0^ upon exposure to AgNO_3_ solution. These silver seeds act as robust catalytic and adhesion sites for subsequent copper growth. Using this approach, we demonstrate circuit traces as narrow as 0.7 µm, featuring excellent uniformity and adhesion. Compared to conventional redistribution-layer (RDL) and under-bump-metallization (UBM) techniques, this process eliminates multiple lithographic and vacuum-based steps, significantly reducing process complexity and production time. The method is scalable and adaptable for applications in transparent electronics, fan-out packaging, and high-density interconnects.

## 1. Introduction

The demand for high-resolution, high-performance metallization on dielectric substrates is rapidly increasing in applications of transparent conducting electrodes (TCEs), flexible electronics, and advanced semiconductor packaging. Electronics integrated on glass surfaces offer numerous applications, such as OLEDs and LCDs, transparent electrodes like metal micro-mesh for touchscreens (as an alternative to costly ITO electrodes), and indoor lighting solutions [1]. Glass, with its optical transparency, dimensional stability, and excellent dielectric properties, is an attractive platform for next-generation interposers, display-integrated circuitry, and fan-out wafer-level packaging (FOWLP). However, traditional metallization methods for glass, particularly those used for redistribution layers (RDLs) and under-bump metallization (UBM), require complex, multi-step fabrication involving photolithography, vacuum deposition, and etching, which are time- and resource-intensive [1,2,3]. In addition to standard lithography-based techniques, several additive and maskless approaches have emerged as alternatives for direct metal patterning on dielectric substrates. Inkjet printing has gained popularity due to its low-cost, non-contact deposition capability and digital control, making it suitable for flexible electronics and prototyping applications. However, its resolution is typically limited to tens of microns, and achieving reliable metal conductivity often requires post-deposition sintering at elevated temperatures, which may not be compatible with all substrates [1,2]. Aerosol jet printing is an emerging circuit-trace-formation technology currently under extensive investigation. However, it remains at an early stage of technological maturity, with limitations in printing speed and circuit widths currently restricted to tens of micrometers, which makes it unsuitable for practical application [4]. Screen printing, while widely adopted in industrial-scale processes for thick-film metallization, also suffers from resolution constraints (typically >50 µm) and stencil wear over time, reducing its applicability for fine-pitch circuitry [3].

Recently, maskless photolithography systems, such as digital-light-processing (DLP)-based direct patterning, have been developed to bridge the gap between resolution and throughput. These techniques use dynamic digital masks to project patterns onto photoresists, allowing microscale precision without the need for photomasks. Although capable of high fidelity, they still rely on photoresist materials, multiple processing steps (coating, exposure, and development), and post-deposition metallization, which introduces process complexity [5,6].

In comparison to the mentioned techniques, they are limited in terms of plating line width for semiconductor packaging. Photolithographic techniques used for RDL formation typically achieve a minimum trace/space resolution of 5/5 µm [3]. Inkjet-printing techniques are generally suited for wider traces, with resolutions around 20/20 µm [1]. DLP-based systems also do not reach submicron resolution, typically achieving around 7–10 µm.

A powerful technique gaining increasing attention for fine metal patterning is selective surface activation induced by laser (SSAIL) [7,8,9,10,11,12,13,14]. This method involves localized modification of the substrate surface using short or ultrashort laser pulses to create chemically active regions that selectively facilitate electroless metal deposition. SSAIL enables direct-write metallization with exceptional spatial resolution, as the laser can define catalytic zones with submicron precision without the need for masks or resists [14]. The process is material-flexible, functioning effectively on glass, polymers, ceramics, and other non-traditional substrates, making it well-suited for applications in flexible electronics, semiconductor packaging, and integrated photonics, where mechanical and electrical material properties must be carefully balanced.

One of the key strengths of SSAIL is the high adhesion strength (5–32 MPa [14]) achieved between the deposited copper and the laser-activated surface, a factor critical for long-term reliability in high-density interconnect applications. The technology has been widely used in fabricating molded interconnect devices (MIDs), typically employing galvanometric scanners to rapidly raster the laser beam across 3D substrates. However, the working field of such systems is limited by the focal length of the F-theta lens, which also defines the minimum achievable spot size, commonly ranging from 15 to 45 µm (FWHM). This limitation constrains the minimum feature size in traditional SSAIL implementations. In contrast, the method demonstrated in this work, based on Bessel beam laser processing of glass surfaces, overcomes this resolution barrier, achieving submicron circuit features with high fidelity across large areas, while preserving the material-selective metallization capability of the SSAIL concept.

In particular, Bessel beams—due to their non-diffracting, extended focal zone—enable precise, high-aspect-ratio surface modifications with minimal thermal damage [15,16,17]. These laser-induced structures can be engineered to locally alter the surface chemistry of glass, forming reactive sites that act as selective anchors for metal nucleation. In this study, we present a streamlined three-step process for fabricating submicron copper circuit traces on glass substrates. The method includes (1) femtosecond or picosecond laser modification using a Bessel beam, (2) chemical activation via silver nitrate (AgNO_3_) immersion, and (3) electroless copper plating. The laser-induced chemical defects promote the in situ reduction of Ag^+^ ions to metallic Ag^0^ nanoparticles, which serve as catalytic and adhesion sites for subsequent copper deposition. This enables the formation of continuous, conductive copper lines with feature sizes down to 0.7 µm—all without photomasks, vacuum tools, or wet etching. Compared to state-of-the-art RDL and UBM fabrication, this approach significantly reduces the number of processing steps, eliminates the need for lithography and plasma etching, and shortens total production time [18,19,20]. It also allows for digital customization and on-demand layout flexibility, which is advantageous for low-volume or design-variable packaging scenarios. This method offers a powerful platform for additive, high-resolution copper metallization on glass, with direct applications in heterogeneous integration, transparent electronics, and advanced microsystems packaging. Moreover, the ability to directly write high-resolution metal traces on glass opens new opportunities in integrated photonic circuits, where precise alignment of optical and electrical pathways is critical for compact, multifunctional devices.

In this study, we conducted an in-depth investigation of submicron copper trace formation as a function of key Bessel beam laser writing parameters. The resulting trace width was influenced by several factors, including laser pulse energy, repetition rate, beam profile, pulse-to-pulse overlap (pitch), scanning speed, and plating duration. To better understand the mechanism behind the laser-induced selective chemical deposition of copper, we performed X-ray-photoelectron-spectroscopy (XPS) analysis, which provided insight into the surface chemistry and catalytic activation induced by laser exposure. Based on these findings, a physicochemical model was proposed to describe the selective reduction of metal ions and subsequent copper nucleation on laser-modified regions. Finally, a functional electrical demonstrator was fabricated, featuring fine copper traces on glass using the described method, demonstrating its potential for transparent-conductive-electrode applications.

## 2. Materials and Methods

### 2.1. Materials

Borosilicate glass D263 (“Schott”, Jena, Germany) (1 mm thick) was used as a substrate material.

### 2.2. SSAIL Steps

All micro-traces were produced using selective surface activation induced by laser (SSAIL), a process comprising three primary steps: (1) laser surface modification using an ultrashort-pulse laser; (2) chemical activation of the laser-modified areas in a catalytic solution; and (3) electroless autocatalytic copper deposition.

**Figure 1:**
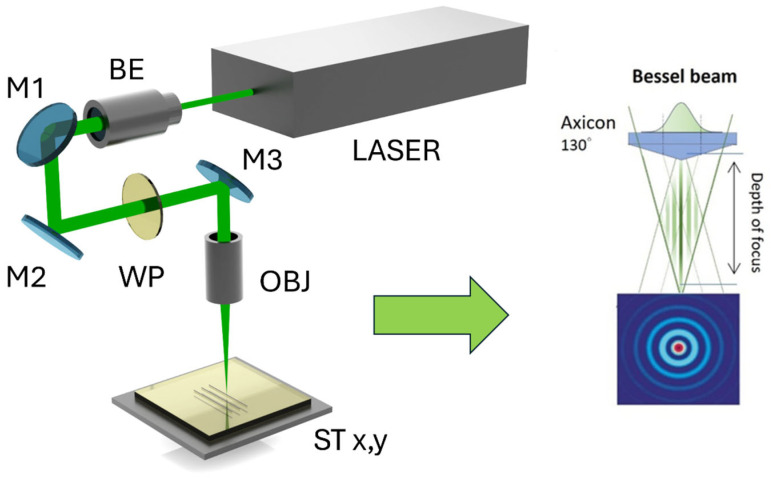
Laser system configuration: BE—beam expander; M1, M2, and M3—dielectric mirrors; WP—waveplate (λ/2); OBJ—objective lens (axicon); ST x,y—linear stages for movement along x and y axes. The Bessel beam schemes also illustrate the focusing depth diagram and the intensity distribution across the beam’s cross section.

Pharos (Light Conversion, Vilnius, Lithuania), a femtosecond solid-state (Yb:KGW) laser with 515 nm wavelength and 10–100 kHz pulse repetition rate, was utilized for laser surface modification. The laser can control its pulse duration. During this experiment, 1 ps and 340 fs pulse durations were investigated. The ANT 130–160 XY (Aerotech, Pittsburgh, PA, USA) linear positioning stage was used for laser beam positioning. An axicon with an apex angle of 130° (Optogama, Vilnius, Lithuania) was used for beam shaping. The input beam diameter was 5 mm. The laser Bessel beam central maximum with an axicon was 2 µm in diameter on the focal plane. The maximum average laser power was 1.3 W, with a maximum pulse energy of 21.47 µJ. Laser beam scanning speed was varied between 0.1 and 300 mm/s. A matrix of laser-modified lines (each 5 mm long) was created to test various processing parameters by varying laser power and scanning speed. During this testing, the scanning speed was increased while simultaneously modifying the pulse repetition rate, ensuring a consistent pulse pitch—the distance between pulse centers along the scanning direction. The key parameters were selected with the aim of achieving the narrowest possible plated line (Figure 1).

**Figure 2:**
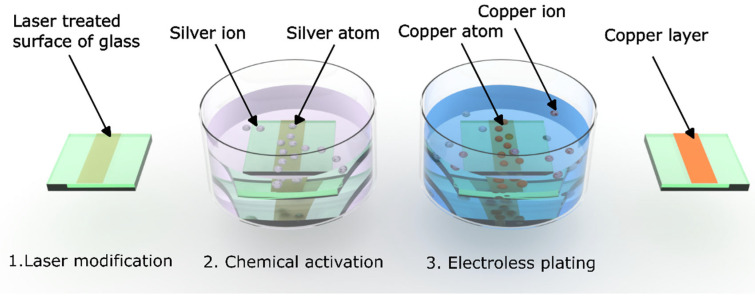
SSAIL process chemical steps.

The dielectric surface requires thorough cleaning and rinsing with distilled water following laser modification. The laser-modified glass sample was immersed in distilled water and placed into an ultrasonic bath at room temperature. After cleaning, the laser-irradiated specimen was activated by immersing it in a silver nitrate (AgNO_3_) solution (Sigma Aldrich, Burlington, MA, USA) with a concentration of 50 µM at 20 °C for 1 min. Subsequently, electroless plating was conducted in a copper plating bath maintained at 50 °C. The bath contained 0.35 M of potassium sodium tartrate, 0.12 M copper sulfate, 1.25 M sodium hydroxide, 0.3 M sodium carbonate, and 3.41 M formaldehyde (Sigma Aldrich, Burlington, MA, USA). The plating durations tested were 10, 20, 25, and 45 min. The chemical steps are represented in Figure 2.

### 2.3. Analysis and Measurements

The scanning electron microscope Helios NanoLab 650 (FEI, Eindhoven, The Netherlands, 2011) dual-beam system with a Schottky-type field emission (FE) electron source, equipped with a gallium ion source (FIB) and an optical microscope (Olympus BX52, Olympus, Tokyo, Japan) with an integrated digital camera, was employed to examine the surface subsequent to the laser irradiation and metallization procedures and cross-sectional analysis. The optical microscope featured objectives with magnification capabilities ranging from 5 to 50×, encompassing both bright and dark field modes of observation. Prior to SEM analysis, samples underwent a coating process utilizing a 50 nm gold layer deposited via the magnetron sputter coater Q150T ES (Quorum Technologies Ltd., Lewes, UK) to prevent any charge buildup on the polymer surface. Evaluation of electrical sheet resistance was conducted employing the four-probe technique [21] using the Keightley 2410 multimeter (Beijing, China). Surface roughness was measured using the profilometer Dektak 150 by Vecco (Sterling Heights, MI, USA). XPS analysis was performed with the Thermo Scientific ESCALAB 250Xi spectrometer equipped with monochromatic Al Kα radiation (hν = 1486.6 eV) (Waltham, MA, USA).

## 3. Results and Discussion

When focusing the Gaussian beam with a lens having a high-numerical aperture of 0.5, the theoretical Rayleigh length $Z_{R}=\frac{\pi {\omega_{0}}^{2}}{\lambda }\approx 17.62$ μm (when w_0 ≈ 1.7 μm), but when using a Bessel beam shape, it is possible to achieve a similar central-lobe diameter while the length of the Bessel zone reaches several tens of mm. This would facilitate surface modification, making it less sensitive to the distance to the sample surface (allowing for processing large material areas). The Bessel beam’s distinctive characteristics play a pivotal role. Due to its non-diffracting nature, the Bessel beam can sustain its energy distribution across longer distances, creating high-aspect-ratio features, precise material ablation, and intricate micromachining in various materials [17]. The unique propagation properties of the Bessel beam enable it to mitigate specific thermal and nonlinear effects, contributing to enhanced processing precision and reduced damage to the surrounding material. Although the central maximum contains only ~20% of the total energy, it is still sufficient to modify the surface. This is due to the energy being concentrated in a very small area, resulting in high energy densities within the central peak. In this study, laser beam shaping with a fused-silica conical-lens axicon was applied. The central maximum of the beam was used for the glass surface modifications.

**Figure 3:**
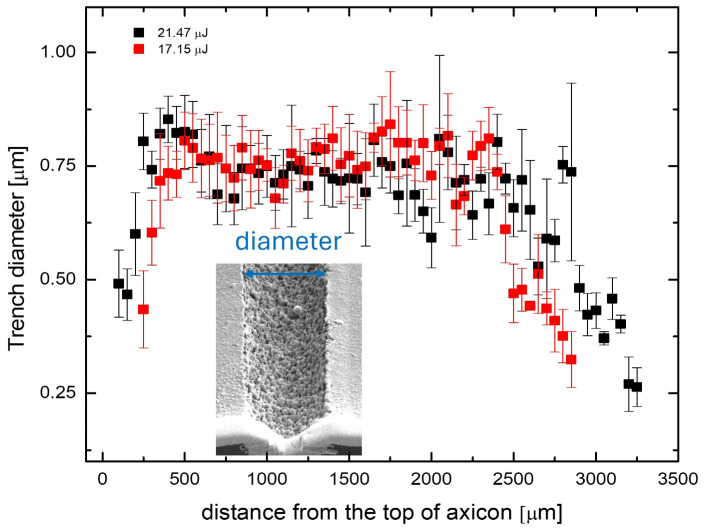
Diameter of the ablated trench as a function of the distance between the axicon apex and the sample surface. The SEM image illustrates the measurement technique.

For this purpose, an axicon was chosen with its apex angle set to 130 degrees—the theoretical central lobe diameter is ~2 μm, and the length of the Bessel zone depends on the size of the beam (the larger the beam before the axicon, the longer the Bessel zone). On the glass surface at different distances from the axicon apex (z = 0), laser writing of lines at 1 mm/s speed and 10 kHz pulse repetition rate was performed to determine the length of the Bessel zone and to observe how the diameter of the damaged zone changed with the distance between the axicon apex and the sample surface (Figure 3). The damage threshold of glass measured >8.60 μJ. Even with the highest pulse energy, the radiation intensity in the ring around the central maximum for glass is insufficient to modify the glass surface. The region where the diameter of the bleached crater remains almost unchanged for glass is quite broad (~2 mm). The depth-of-field characteristic was included to highlight the application flexibility of this approach for industrial use. Unlike Gaussian beam focusing, which has a very narrow Rayleigh length and requires precise focal positioning, this method allows for a significantly larger tolerance in maintaining the focal position. This makes the technology more attractive for large-scale processing applications.

Initially, the writing speed was experimentally set to achieve a pulse pitch of 100 nm, ensuring the formation of a continuous laser-modified line. A smaller pitch was avoided due to the risk of thermal damage and microcracking in the glass, caused by local heat accumulation from overlapping pulses. On the other hand, increasing the pulse pitch can lead to discontinuities in the laser-induced modification, which results in breaks in the continuity of the subsequently plated copper traces.

**Figure 4:**
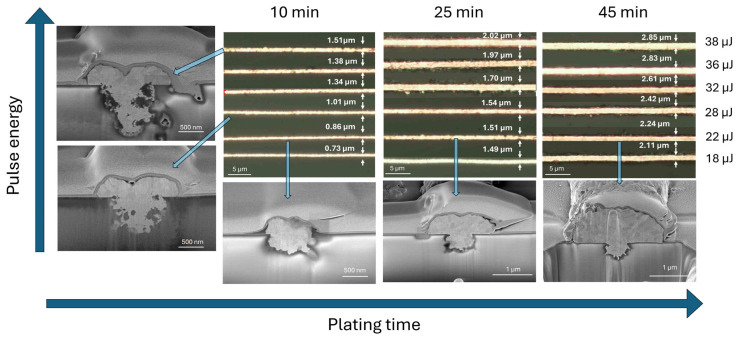
Microscope images of deposited copper traces with measured widths for different laser pulse energies and plating times. Cross sections of selected traces, prepared using FIB, are indicated by arrows for pulse energies of 38, 28, and 22 µJ and plating times of 10, 25, and 45 min. Laser pulse repetition rate was 10 kHz.

Copper was electrolessly deposited (following activation process) onto the laser-modified lines using varying immersion times in the copper plating bath. The resulting trace widths were characterized using optical microscopy. As shown in Figure 4, the width of the plated copper traces depended on both the laser pulse energy and the duration of the plating process. At the lowest laser pulse energy and shortest plating time, the initial trace width was approximately 700 nm. As the pulse energy increased, the width of the copper traces expanded, reaching up to 1.5 µm. For extended plating durations of up to 45 min, the trace widths further increased—from approximately 2.0 µm at 18 µJ to 2.4 µm at 38 µJ—indicating that both laser energy and plating time contribute cumulatively to the final feature size. This means that the final line dimensions can be effectively tuned by adjusting the laser energy or the plating time. Focused-ion-beam (FIB) cross-sectional analysis (see Figure 4) revealed distinct mechanisms behind trace broadening in each case.

At higher laser pulse energies, the copper follows the geometry of a broader, more deeply ablated trench, which results from increased energy density during laser exposure. Conversely, when the plating duration is extended, copper grows beyond the boundaries of the laser-modified zone, expanding laterally due to the isotropic nature of electroless deposition. This behavior is consistent with prior reports indicating that electroless copper nucleates uniformly around catalytic seed sites and grows in all directions under stable bath conditions [21,22]. Furthermore, FIB imaging revealed that higher pulse energies (e.g., 38 µJ) can induce subsurface damage in the glass substrate, including forming voids and microcracks—likely caused by stress relaxation following localized melting and rapid cooling. In contrast, no such defects were observed for lower pulse energies in the range of 18–28 µJ, indicating that this energy range provides sufficient surface activation without compromising structural integrity.

To achieve greater trace thickness, extended copper plating times are required. These findings are essential for optimizing the patterned features’ resolution and mechanical robustness.

**Figure 5:**
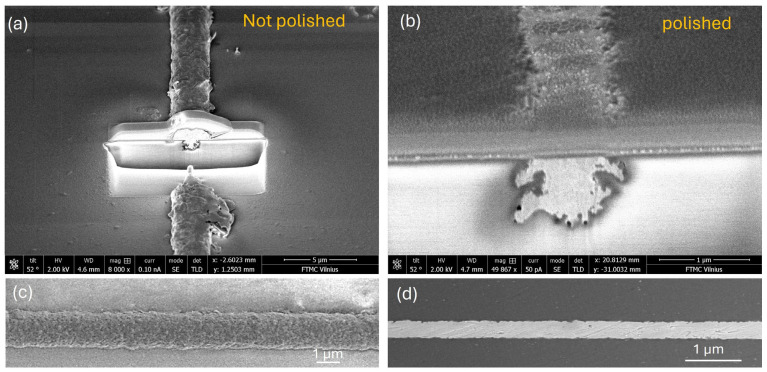
SEM images of copper trace fabricated using 28 µJ laser pulse energy and 25 min of plating in electroless bath: (**a**) cross section after plating, (**b**) cross section after polishing, (**c**) SEM image of copper trace after plating, and (**d**) SEM image of trace after polishing.

In a subsequent experiment, copper traces fabricated with various pulse energies and plated for 20 min were subjected to a post-processing step using a zirconium-based prophylaxis polishing paste with sub-2 µm grain size. The objective of this test was to improve edge smoothness and enhance dimensional uniformity across the trace. Cross-sectional and SEM images of a representative trace processed with 26 µJ pulse energy and plated for 20 min are shown in Figure 5, both before and after polishing. The images reveal that polishing significantly reduces the overall trace width, conforming it more closely to the original laser-modified trench dimensions (Figure 5b). Additionally, a more embedded and uniform copper structure is achieved (Figure 5c,d) within the trench. However, a concurrent reduction in trace thickness is also observed due to the polishing process (Figure 5a).

**Figure 6:**
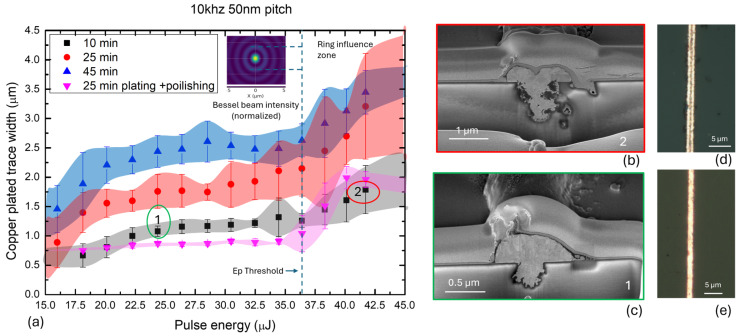
(**a**) Copper trace width as a function of laser pulse energy for different plating durations (10, 25, and 45 min), including standard deviation bands as a visual guide. A threshold behavior is observed near 37 µJ (indicated 2 in the graph (**a**)). (**b**,**d**) Cross-sectional and optical microscope images of a copper trace formed above the threshold energy, showing dual growth initiation zones attributed to the Bessel beam’s second-order ring. (**c**,**e**) Corresponding cross-sectional and optical images of a trace formed below the threshold, exhibiting a single growth zone confined to the central trench (indicated 1 in the graph (**a**)).

All measured trace widths, along with their dependence on laser pulse energy and plating time, including standard deviation (STD) values, are presented in Figure 6. The data reveals a general trend of increasing trace width with rising laser pulse energy. This trend is pronounced for samples subjected to longer plating times. In contrast, the trace width remains almost constant for polished samples up to a pulse energy of 36 µJ, indicating that polishing effectively removes the excess copper grown beyond the laser-modified trench boundaries. Standard deviation values are higher for longer plating durations, reflecting increased variability in lateral copper growth. These deviations, visualized as shaded color bands in Figure 6, serve as a guide for the eye and highlight the precision of trace width control. For extended plating times (25–45 min), the variability in width ranges from approximately 0.3 to 0.6 µm. This increased fluctuation may be attributed to instabilities in the electroless-copper-growth process during prolonged deposition, as reported in prior studies on isotropic plating dynamics [5,23]. In contrast, the standard deviation is minimal for polished samples, indicating a high degree of uniformity once surface excess is removed. A notable observation is the sudden increase in trace width when the laser pulse energy exceeds approximately 37 µJ—identified as a threshold energy Ep, marked in Figure 6. This sharp transition occurs across all plating durations and is likely associated with the emergence of secondary energy deposition effects due to the Bessel beam’s second-order ring. As illustrated in Figure 6b,d, copper deposition appears to initiate from two distinct zones located symmetrically around the trench center, separated by approximately 1 µm. This spacing corresponds to the calculated diameter of the Bessel beam’s second-order ring (see Equation (1)), suggesting a direct correlation. Such a dual-seeding pattern is not observed in traces formed below the threshold energy, as seen in Figure 6c,e. We define this region of dual initiation as the ring influence zone, indicating a critical regime where the outer rings of the Bessel beam begin to participate in substrate modification and catalytic activation actively.

The central radius *r*_0_ of a Bessel beam can be estimated using the following relation:

(1) $$r_{0}=\frac{0.38\cdot \lambda }{sin(\Theta )}\;\;\;where\;\;\;\;\;\Theta \approx \left(n-1\right)\alpha \;\;and\;\alpha =90^{o}-\frac{A}{2}$$

In this expression, *r*_0_ is the central radius of the Bessel beam, *λ* is the wavelength, *α* is the base angle of the axicon, *A* is the physical apex (or axicon) angle, *Θ* is the resulting Bessel beam cone angle, and n is the refractive index of the axicon material (fused silica, in this case). Using this relationship, *r*_0_ evaluates to approximately 1.8 µm for the given parameters. These definitions form the basis for understanding and designing optical systems that utilize Bessel beams, including laser micromachining, optical trapping, and extended-depth imaging applications.

**Figure 7:**
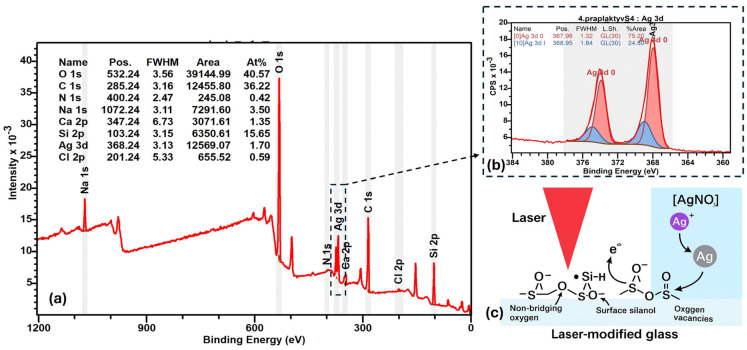
XPS spectra of glass samples lasered and activated: broad spectra (**a**), Ag3d bands range, where the red area in the XPS spectrum of silver represents the fitted peak corresponding to the atomic state of silver (Ag^0^), while the blue area represents the fitted peak of the ionic state of silver in the analyzed spectral data (**b**), and scheme representing activation mechanism (**c**).

The surface chemical composition analysis was performed using X-ray photoelectron spectroscopy (XPS) to investigate the changes induced by laser processing followed by AgNO_3_ immersion. The comparison between non-lasered and lasered glass surfaces revealed significant compositional shifts (Figure 7a). After laser treatment, a slight increase in carbon content was observed, from 23.7% to 25.4%, suggesting the formation of new carbonaceous groups on the surface, potentially acting as mild reducing agents for Ag^+^ ions [24]. Simultaneously, a notable increase in sodium content from 3.2% to 6.7% was detected, indicating sodium migration towards the surface due to localized heating and structural rearrangements induced by the ultrashort laser pulses [2]. Conversely, the concentrations of nitrogen, oxygen, and silicon slightly decreased. Nitrogen content dropped from 0.25% to 0.17%, oxygen content reduced from 50.8% to 49.5%, and silicon content declined from 20.9% to 17.1%, indicating a partial depletion of the native glass network at the surface. These changes confirm that laser irradiation significantly modifies the glass surface chemistry, favoring conditions for the subsequent reduction of silver ions and the deposition of metallic silver nuclei, which act as catalytic centers for electroless copper plating.

### Defect-Mediated Reduction of Ag^+^ on Ultrafast-Laser-Processed Glass Surfaces

Ultrashort-pulse-laser irradiation (fs regime) induces localized chemical and structural modifications in glass, enabling surface-driven reduction of silver ions. X-ray photoelectron spectroscopy (XPS) reveals that ~75% of the silver present on laser-treated surfaces is in the metallic state (Ag^0^) (see Figure 7b), confirming an efficient reduction process. This behavior is attributed to the formation of non-bridging oxygen sites, oxygen vacancies, and silanol groups (≡Si–OH) during laser exposure, which act as electron-donating centers facilitating Ag^+^ → Ag^0^ reduction. Upon immersion in AgNO_3_ solution, adsorbed Ag^+^ ions are spontaneously reduced via defect-mediated electron transfer, forming Ag^0^ nanoparticles in the absence of external reducing agents (please see visual explanation in Figure 7c). Similar mechanisms involving laser-induced free-electron generation and defect formation have been reported in silver-containing phosphate and silicate glasses under ultrafast-laser exposure [24,25,26,27,28]. The increased Na content observed via XPS suggests ion migration from the glass bulk, which may further influence local charge compensation during silver deposition. These results support a model wherein laser-activated glass surfaces provide localized redox functionality, enabling spatially selective silver nanoparticle formation through purely photonic processing. Together, these observations support a model in which ultrafast-laser processing generates surface states capable of driving Ag^+^ → Ag^0^ transformation via direct electron transfer.

**Figure 8:**
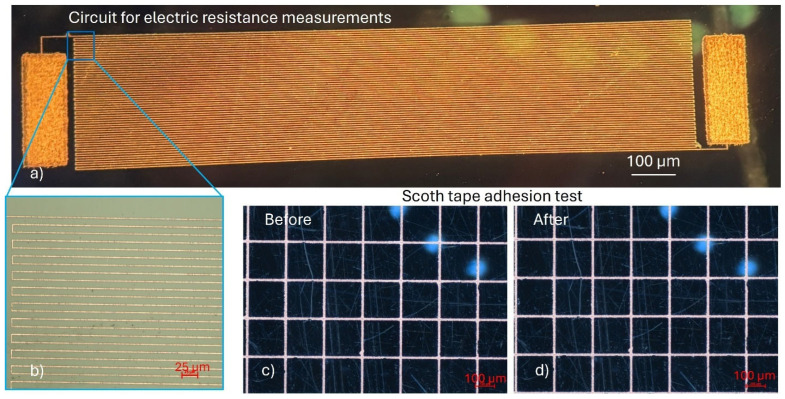
Copper circuit structures fabricated for electrical and adhesion tests: (**a**) optical image of the meander structure used for electrical conductance measurements; (**b**) optical microscope image of a meander segment; (**c**) optical image of the copper mesh structure on glass before the adhesion (Scotch tape) test; and (**d**) optical image of the mesh after the adhesion test.

The electrical and mechanical properties of the deposited copper structures were evaluated to assess their functionality and integration potential. For electrical conductivity measurements, a copper meander line was fabricated on a D263 glass substrate, featuring a trace width of 2.5 µm and a total length of 60 mm (Figure 8a,b). The measured electrical resistance of the structure was 735 Ω with a standard deviation of 9.5 Ω. This value represents less than 30% deviation from the theoretically (550 Ω) calculated resistance, confirming the high conductivity and uniformity of the deposited copper. Additionally, the surface roughness of the sample was measured using a mechanical profilometer, showing a Sa value of 0.34 µm for the plated copper.

Adhesion of the copper to the glass substrate was assessed using a standard Scotch tape test following ASTM D3359 guidelines [8,29]. For this purpose, a copper mesh structure was patterned onto the glass surface (Figure 8c,d). After performing the tape test, no detachment or delamination of the copper was observed, corresponding to an adhesion classification of 4A, indicating excellent adhesion properties.

The excellent adhesion of electrolessly deposited copper observed in this study is attributed to the synergistic effects of chemical bonding, direct in situ nucleation, and mechanical interlocking at the interface between laser-reduced Ag^0^ nanoparticles and the glass substrate. Ultrashort-pulse-laser irradiation introduces a high density of surface defects, including oxygen vacancies and hydroxyl groups, which serve as energetically favorable nucleation sites. During immersion in the AgNO_3_ solution, Ag^0^ atoms are reduced from Ag^+^ ions and directly anchored onto these defect sites, possibly forming strong chemical bonds such as Ag–O and Ag–Si–O linkages (see Figure 8c), thereby ensuring robust initial attachment of silver to the glass surface [24]. In addition to chemical anchoring, the silver nucleates directly onto the laser-modified substrate without the presence of an intermediate barrier layer, as typically found in conventional physical deposition techniques like sputtering or evaporation. This direct nucleation ensures intimate atomic-scale contact at the silver–glass interface, promoting superior adhesion. Moreover, the ultrashort-pulse-laser treatment induces microscale surface roughening, creating a textured morphology that enhances mechanical interlocking between the growing silver nanoparticles and the substrate.

Together, these effects establish a highly stable silver seed layer that is an efficient catalyst for subsequent copper deposition. The atomic continuity and strong interfacial interactions at the Ag–Cu interface ultimately led to excellent adhesion strength of the final electrolessly plated copper, as demonstrated by the mechanical and electrical tests conducted in this work.

**Figure 9:**
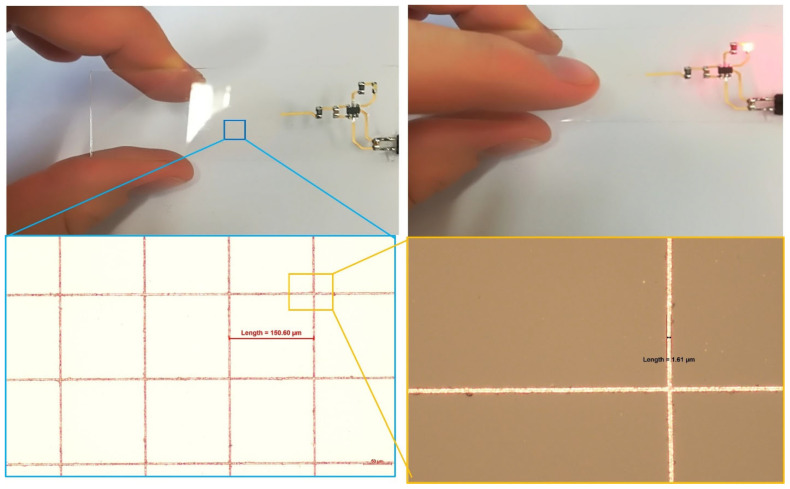
Demonstration of functional copper circuit fabricated on glass: optical images of the transparent capacitive touch sensor based on copper mesh structures, where touching the glass surface activates an LED indicator.

Finally, the functionality of the fabricated structures was demonstrated through a transparent electronic demonstrator. A capacitive touch “button” was implemented by patterning a transparent copper mesh on the glass surface. Upon touch activation, an LED light was triggered, verifying the electronic performance of the patterned copper structures (Figure 9).

## 4. Conclusions

This investigation provides substantial progress in the understanding and application of ultrafast-laser-induced surface modifications on glass for nanoscale copper patterning. Employing femtosecond Bessel beams followed by a chemical activation process with silver ions and electroless copper plating, we successfully fabricated highly uniform copper features as narrow as 0.7 µm. Comprehensive characterization via X-ray photoelectron spectroscopy elucidated the pivotal role of laser-generated surface defects, particularly non-bridging oxygens and silanol groups, in promoting the reduction of silver ions. These defects serve as catalytic nucleation centers, ensuring high-density, adherent copper growth strictly confined to irradiated areas. The robustness of the copper–glass interface, demonstrated through rigorous mechanical adhesion tests and practical electronic-device implementation (capacitive touch sensor), emphasizes the method’s reliability and scalability. This research not only advances fundamental knowledge of defect-mediated metal deposition at the nanoscale but also highlights significant implications for emerging applications in transparent nanodevices, integrated photonic circuitry, and high-density microsystems packaging, marking a valuable contribution to the field of laser-driven nanomaterial processing.

## Data Availability

The datasets used and/or analyzed during the current study are available from the corresponding author on reasonable request.
